# Functions, Features, and Psychological Well-Being Impacts of Type 1 Diabetes Self-Management Mobile and Web Apps: Systematic Review

**DOI:** 10.2196/75280

**Published:** 2025-11-19

**Authors:** Titouan Cloarec, Katie Cunneen, David Nickson, Simon Leigh, Petra Hanson, Carla Toro

**Affiliations:** 1 Warwick Applied Health, Warwick Medical School, University of Warwick, Gibbet Hill Campus, Coventry, CV4 7AL, United Kingdom, 44 07491330344; 2 Department of Psychology, University of Warwick, Coventry, United Kingdom

**Keywords:** type 1 diabetes, psychological well-being, mobile health, web-based interventions, PRISMA

## Abstract

**Background:**

People living with type 1 diabetes must adhere to an intense self-care regimen, which may impact their psychological well-being and contribute to poor self-management behaviors. Despite their potential, most mobile health and web-based apps for diabetes management prioritize glycemic control and often overlook psychological well-being. As a result, evidence on the effectiveness of these interventions in improving psychological well-being remains limited, and there is still uncertainty about which functions and features are the most effective.

**Objective:**

The objective of this review was to assess changes in the psychological well-being of people with type 1 diabetes and identify the functions and features of mobile and web-based interventions that may enhance their psychological well-being.

**Methods:**

Relevant studies were identified through PubMed, Web of Knowledge, Embase, Scopus, APA PsycInfo, and the Cochrane Central Register of Controlled Trials, with the search conducted at the end of November 2024. Studies were included if they quantitatively assessed the impact of mobile health or web-based apps on psychological well-being in people with type 1 diabetes using validated screening tools. A conventional content analysis approach was used to categorize the functions and features of the included interventions.

**Results:**

In total, 8 of the 2142 articles identified met the inclusion criteria and were included in the review. Six categories of functions were identified, each incorporating different sets of features: (1) therapy, (2) education, (3) self-management, (4) peer support, (5) health care professional–patient support, and (6) parental support. Only 2 of the 8 studies reported improved psychological well-being. One of these 2 studies included therapy-based interventions, while the other combined self-management, education, and peer support functions. However, the limited number of studies and variability in study design and participant characteristics limited the ability to attribute the effectiveness in improving psychological well-being to specific functions and features or their combinations.

**Conclusions:**

This review highlights the limited effectiveness of currently available mobile health and web-based interventions in improving the psychological well-being of people living with type 1 diabetes. While some interventions showed promise, the findings highlight the need for targeted, theory-based approaches; stakeholder involvement in intervention design and development; and combination of functions and features to improve support and long-term outcomes.

## Introduction

Type 1 diabetes, an autoimmune disease, affected approximately 9 million people in 2022, of whom 1.52 million were younger than 20 years [1]. It is characterized by an immune-mediated depletion of insulin-producing beta cells, resulting in a lifelong dependence on exogenous insulin [2]. People living with type 1 diabetes are therefore required to have an intense self-care regimen involving daily insulin administration, glucose monitoring, carbohydrate counting, and physical activity [3]. Adherence to this self-care regimen is essential, as prolonged hyperglycemia (high blood glucose levels) can lead to severe and potentially life-threatening diabetes-related complications [4].

The demanding self-care regimen for people living with type 1 diabetes is thought to contribute to the higher prevalence of psychological well-being problems compared to the general population [5,6]. These include diabetes distress, depression, anxiety, and eating disorders [7]. For example, 1 in 3 people living with type 1 diabetes experience substantial diabetes distress [8]. Furthermore, people with type 1 diabetes have a significantly higher prevalence of depression than the general population (22% vs 13%) [9,10], which further highlights the significant psychological challenges they experience. These psychological challenges are strongly associated with poor self-management behaviors, such as less frequent blood glucose monitoring and suboptimal glycemic control. In recognition of this, psychological well-being has become a recognized priority in diabetes care [11] and has led to the development of targeted psychological interventions to support patients with the unique challenges of living with diabetes.

Studies evaluating psychological interventions such as cognitive behavioral therapy (CBT) or guided self-determination demonstrate subtle clinical benefits in type 1 diabetes management [12]. Although these interventions were not found to be associated with changes in glycemic control, they enhanced self-reported patient functioning, reduced diabetes-related distress, and improved psychological well-being [12]. Mindfulness-based cognitive therapy and self-determination theory have also been shown to improve psychological well-being in people living with diabetes [13,14]. This highlights the potential of targeted psychological treatments to support the mental well-being of people managing type 1 diabetes. However, psychological therapies are not part of standard care [15] due to various reasons, including limited availability of health care professionals (HCPs); challenges in scaling these programs to all patients in need; and the significant logistical and time requirements for HCPs, families, and patients [15,16].

Digital health interventions via mobile health (mHealth) and web-based apps offer the opportunity for patients with type 1 diabetes to manage their condition at minimal costs [17]. Most type 1 diabetes mHealth app studies to date have primarily focused on glycemic control as the primary outcome, with limited focus on psychological well-being [18,19].

Knox et al [20] conducted a systematic review to assess the effectiveness of digital health interventions in improving physical health, psychological well-being, and cognitive outcomes in children and young people with type 1 diabetes. Among the 30 studies included, only 4 of the 10 that assessed psychological or cognitive outcomes reported improvement; however, these outcomes focused primarily on self-efficacy and quality of life rather than on specific measures of psychological well-being [20]. The interventions in these studies included text messaging, games, and a training program for self-management. Similarly, Garner et al [21] reviewed 15 studies on digital interventions for psychological well-being in youths aged 5‐25 years and found improvements in self-efficacy through gaming and self-management apps but no consistent overall benefits for psychological well-being.

Within both reviews, studies demonstrating improvements largely focused on self-efficacy and quality of life rather than directly addressing psychological well-being itself. While self-efficacy and quality of life are valuable outcomes, they can be influenced by a range of factors beyond psychological well-being [20,21]. Therefore, it is important to consider psychological well-being as a distinct entity to identify interventions that can directly address and improve the psychological challenges associated with living with type 1 diabetes.

Overall, these previous reviews have highlighted that the current digital interventions, particularly mHealth apps, fail to address psychological well-being effectively, despite its important role. As established in Garner et al [21], there is a lack of consistent evidence for improvement in psychological well-being in young people with type 1 diabetes. This highlights the need to identify the specific functions—that is, the main purpose—and their features (ie, the components or elements of an intervention designed to carry out the specific functions) to improve the psychological well-being of people with type 1 diabetes.

Building on the findings of Knox et al [20] and Garner et al [21], this review aims to provide an updated synthesis of the current evidence focusing specifically on mHealth and web-based interventions for both children and adults with type 1 diabetes. This review assesses changes in the psychological well-being of people with type 1 diabetes and identifies the functions and features of mHealth and web-based apps that may underpin improvements in psychological well-being.

## Methods

This systematic review was conducted according to the PRISMA (Preferred Reporting Items for Systematic Reviews and Meta-Analyses) guidelines (Checklist 1). The protocol for this review was registered in the international prospective register of systematic reviews (PROSPERO), an online database for systematic review protocols (CRD42024509788) [22,23].

### Search Strategy

The databases Embase, PubMed, the Cochrane Central Register of Controlled Trials, Scopus, Web of Science, and APA PsycInfo were searched for relevant peer-reviewed articles on November 26, 2024. Search terms and their synonyms and variations were categorized by population (type 1 diabetes), intervention (mHealth and web-based apps), and outcomes (psychological well-being). In addition, the search terms were developed with the assistance of a research and academic support librarian. The search strategy included controlled vocabulary, such as MeSH terms for PubMed, and free-text vocabulary. Keywords and terms were combined using Boolean operators (AND and OR). Searches were regularly checked to ensure that the outcomes were relevant. A preliminary search was conducted to ensure that relevant articles were captured before proceeding. The search strategy is detailed in Table S1 in the Multimedia Appendix 1. References cited in previously published systematic reviews and the included studies were manually searched to identify additional papers.

### Eligibility

Studies were included if they (1) were available in English, (2) involved people with type 1 diabetes aged 10 years and older using mHealth apps, and (3) quantitatively assessed the impact of the mHealth intervention on psychological well-being using validated screening tools such as the Problem Areas in Diabetes (PAID) scale or Diabetes Distress Scale (DDS). Eligible study designs included single-arm studies, before-and-after studies, randomized controlled trials (RCTs), observational studies, and quasi-experimental designs. The age of 10 years was chosen, as the peak age at diagnosis for type 1 diabetes is between 10 and 14 years [24].

Studies were excluded if they (1) were systematic reviews or meta-analyses; (2) were qualitative studies; (3) had incomplete information; (4) included participants younger than 10 years; (5) included participants with type 2 diabetes, gestational diabetes, or prediabetes or did not separate the results for type 1 diabetes and type 2 diabetes (in studies involving both types); or (6) focused on other forms of digital health (eg, wearable devices or telehealth, including virtual consultations).

### Study Selection

Selected articles from database searches were uploaded to Rayyan (Rayyan Systems, Inc) [25], a software used for systematic reviews, and duplicates were removed. The abstracts and titles of all studies were independently reviewed by 2 authors (TC and KC) using Rayyan. Discrepancies were resolved by discussion. In case of disagreement, a third reviewer (PH) would review the articles and determine whether they should be included for the full-text review.

The full text of the articles was reviewed by 1 review author (TC), with a secondary reviewer (KC) screening 20% of all articles [26]. In case of disagreement, a third reviewer (DN) reviewed the articles and determined if they should be included.

### Data Extraction

Data were extracted by 1 review author (TC), and a random 20% sample was independently extracted by a second author (KC) into an Excel spreadsheet. The following information was extracted from the articles (see Tables S2a-f in Multimedia Appendix 1): year of study, study design, population characteristics, mHealth and web-based app functions and features, and outcome measures (psychological and physical)

### Data Synthesis

To categorize the functions and features of the interventions that we included in the review, a conventional content analysis approach was used. First, a detailed description of the interventions and their included features was extracted from the articles and from within any available supplementary material. An open coding process was applied by one researcher (TC), where each feature was labeled based on its function or purpose within the intervention.

After coding, the labeled features were examined to identify patterns or common functions across the studies. This involved grouping related features into broader categories. For example, all features related to the management of type 1 diabetes (eg, carbohydrate counting and alarms for self-management tasks) were grouped under the “self-management” function.

The categories were then refined to accommodate all features from the included studies and ensure that each feature was appropriately classified. Function categories and included features were then reviewed by 2 separate authors (CT and PH), with modifications made through discussion where necessary.

### Risk of Bias Assessment

One author (TC) assessed the risk of bias in the included studies using the Risk of Bias 2 (RoB 2) tool for RCTs and the Risk of Bias in Non-randomised Studies - of Interventions (ROBINS-I) tool for nonrandomized studies [27,28]. The RoB 2 tool assesses 5 domains of bias, categorizing each as “low risk,” “some concerns,” or “high risk” following a predefined algorithm, to determine an overall risk of bias score. Similarly, the ROBINS-I tool evaluates 7 domains, rating each as having low, moderate, serious, or critical risk of bias, with an overall score derived from these ratings. A 20% sample of the included studies was independently assessed by a second author (KC).

## Results

### Study Selection

**Figure 1.**
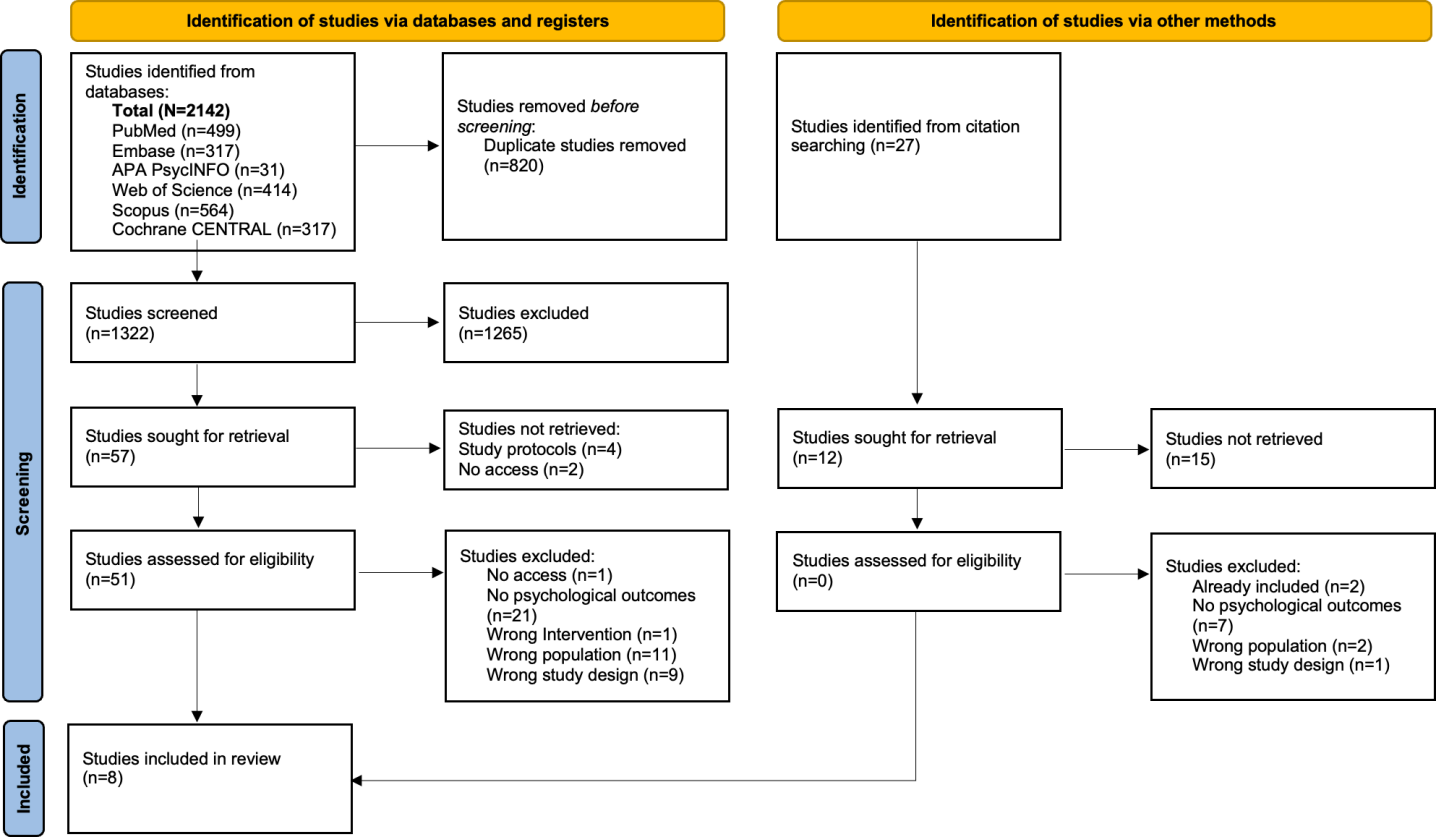
PRISMA (Preferred Reporting Items for Systematic Reviews and Meta-Analyses) flow diagram summarizing the literature search and article selection process.

Figure 1 illustrates the literature search and selection process. The database search yielded a total of 2142 articles. Of these, 51 were retained for a full-text review after duplicates were removed. Following a full-text review, 8 studies were included.

In total, 43 articles were excluded for the following reasons: (1) the full text was not accessible; (2) the study did not report psychological well-being; (3) the study did not use an mHealth or web-based app; (4) study participants with type 1 diabetes and type 2 diabetes were not separated in the Results section, or the mean participant age was below 10 years; or (5) the study was qualitative without a quantitative element. The study design and participant characteristics of the included studies are summarized in Table 1.

**Table 1.** Detailed study characteristics and participant characteristics of the included studies.

Study	Aim	Intervention type	Study design	Participant characteristics	Sample size	Age (years), mean (SD)	Female participants, n (%)	Diabetes duration (years), mean (SD)
Carreira et al (2023) [29]	To administer an internet-based CBT^a^ program for the treatment of mild-moderate depressive symptomatology in individuals with T1D^b^ and to evaluate the efficacy of this program	Web	RCT^c^	Adults with T1D (aged ≥18 years); mild to moderate depressive symptoms	65	Treatment group: 37.5 (11.0); control group: 35.0 (12.9)	38 (58)	Treatment group: 18.7 (12.7); control group: 21.3 (12.8)
Tack et al (2018) [30]	To evaluate a prototype integrated mobile phone diabetes app in people with T1D	Mobile	Cohort	Adults with T1D (aged ≥18 years); diabetes duration (≥2 years)	19	43.8 (14.1)	12 (63)	22.8 (14.0)
Singh et al (2023) [31]	To assess the efficacy of an internet-based CBT intervention in adolescents with T1D and depressive symptoms	Web	Cohort	Adolescents with T1D (aged 13‐17 years); mild or moderate depressive symptoms	7	15.1 (1.2)	6 (86)	6.0 (3.4)
Drion et al (2015) [32]	To investigate whether the use of a digital diabetes diary results in a change in quality of life for patients with T1D compared with the standard paper diary	Mobile	RCT	Adults with T1D (aged ≥18 years)	63	33.0 (21)^e^	23 (37)	17.0 (16.0)^e^
Hilliard et al (2020) [33]	To evaluate the feasibility and acceptability of a new, strengths-based mHealth^d^ app for parents of adolescents with T1D	Mobile	RCT	Adolescents with T1D (aged 12‐17 years); diabetes duration (≥6 months)	80	15.3 (1.5)	47 (59)	5.7 (3.4)
Castonsøe-Seidenfaden et al (2018) [34]	To assess the feasibility and acceptability of a mobile-friendly web app aimed at enhancing self-management among young people	Web	RCT	Individuals with T1D (aged 4‐22 years); T1D for at least 1 year	151	17.6 (2.6)	81 (54)	8.0 (4.5)
Xie et al (2023) [35]	To evaluate user satisfaction with a web application and investigate changes in the self-reported frequency of and fear of hypoglycemia and diabetes-related self-efficacy	Web	Mixed methods	Adults with T1D (aged ≥18 years)	207	49.3 (13.8)	135 (65)	25.2 (14.7)
Cuixart et al (2024) [36]	To examine whether an app for diabetes management, together with teleconsultations, can have a positive impact and replace current clinical care	Mobile	RCT	Adults with T1D (aged ≥18 years); diabetes duration (≥1 year)	25	44.5 (14.8)	12 (48)	21.3 (14.1)

a CBT: cognitive behavioral therapy.

b T1D: type 1 diabetes.

c RCT: randomized controlled trial.

d mHealth: mobile health.

e Reported as median (IQR).

### Characteristics of Included Studies

#### Study Design and Duration

The 8 included articles were published between 2015 and 2024. Included studies were published in the United States [31,33], the Netherlands [30,32], Spain [29,36], Denmark [34], and Canada [35].

The selected studies included 5 RCTs [29,32-34,36], 2 cohort studies [30,31], and 1 mixed methods study [35]. The intervention duration ranged from 6 weeks [30] to 12 months [34-36], with 2 studies lasting less than 3 months [29,30] and the remaining 6 lasting more than 3 months. All 5 RCTs reported participant randomization, but 2 of them did not clearly specify how randomization was conducted [33,34].

#### Participant Characteristics

The studies included participants with a mean age ranging from 10 to 49 years. Of these, 5 studies included adults with type 1 diabetes (aged ≥18 years) [29,30,32,35,36], and 3 studies included participants aged 10 to 18 years with type 1 diabetes [31,33,34]. The sample size of the different studies ranged from 7 participants [31] to 207 participants [35], with a total of 617 participants across all 8 studies. Notably, 57% (354/617) of participants across all studies were female [31]. The diabetes duration of participants ranged from a mean of 5.7 (SD 3.4) years [33] to 25.2 (SD 14.7) years [35].

#### Participant Recruitment

Of the 8 studies, 6 studies used routine clinical visits to recruit participants [29-33,36]. In the other 2 studies, participants were recruited through a registry of people with type 1 diabetes [35] and via telephone calls [34], respectively.

### Features and Functions

The functions within the mHealth and web-based interventions investigated in each study were divided into the following categories: (1) therapy, (2) education, (3) self-management, (4) peer support, (5) HCP-patient support, and (6) parental support. The function categories, including the various features within each intervention, are outlined in Table 2.

**Table 2.** Categories of functions with the included features within mobile health and web-based apps.

Study	Therapy	Education	Self-management	Peer support	HCP^a^-patient support	Parental support
Carreira et al (2023) [29]	Includes 9 weekly sessions based on CBT^b^, covering topics such as depression, diabetes distress, diabetes, and coping in diabetes, among others	—^c^	—	—	—	—
Tack et al (2018) [30]	—	—	Logbook: record key diabetes metrics. Carbohydrate tracking: save standard meals and find the carbohydrate content. Custom settings: adjust target glucose levels, set alarms, receive bolus advice, and enable warnings. Bolus advice: Receive insulin dosing recommendations based on personal data and carbohydrate to insulin ratios.	Online community to connect with peers	Communicate with HCPs via messages through a secure connection	—
Singh et al (2023) [31]	Includes 14 online self-directed modules incorporating CBT, BA^d^, and IP^e^	—	—	—	—	Information for parents to support the adolescent with T1D
Drion et al (2015) [32]	—	—	Enter diabetes-related self-care data: blood glucose values, carbohydrate intake, medication, physical exercise, and notes	—	—	—
Hilliard et al (2020) [33]	—	3 brief videos featuring a psychologist (for parents)	—	—	—	List of 16 strength behaviors for parents to mark if their teenaged child engaged in a strength behavior that day, plus a free text option; list of 3 most frequent strengths of their teenaged child over the previous week; list of messages for parents to copy and paste into a text message and personalize to praise their teenaged child
Castonsøe-Seidenfaden et al (2018) [34]	—	—	Information and tips: covers carbohydrate counting, T1D^f^ topics (eg, driver’s licenses), and hypoglycemia tips. Reminders: set alerts for self-management tasks	Chat room to chat with peers	Enables users to contact their HCP	Provides parents with information about how to support their teenaged child
Xie et al (2023) [35]	—	6 categories of learning modules with several courses in each; contains videos, quizzes, testimonials, and PDFs	Automated carbohydrate counter	Discussion forum: all users share the same discussion forum moderated daily by the support coordinator. Users can post in the language of their choice and “like” or respond to other posts.	—	—
Cuixart et al (2024) [36]	—	—	Diabetes diary with automatic collection and analysis of data on glycemia (including estimation of HbA1c^g^) and the possibility to manually enter information on food intake, physical activity, and insulin dose; a bolus calculator; reminders about blood glucose monitoring; possibility to save pictures of food consumed	—	—	—

a HCP: health care professional.

b CBT: cognitive behavioral therapy.

c Not applicable or not available.

d BA: behavioral activation.

e IP: interpersonal psychotherapy.

f T1D: type 1 diabetes.

g HbA1C: glycated hemoglobin.

Of the 8 studies, 2 included therapy as a core function (Table 3). These included features involving 8-10 self-directed modules focusing on CBT [29,31], behavioral activation [29], and interpersonal therapy [31].

Two studies [33,35] incorporated educational functions. One mHealth intervention used videos to educate parents of teenagers with type 1 diabetes on the importance of praising their teenaged children [33], while the other intervention focused on general type 1 diabetes self-management [35]. The second study also included interactive features, such as quizzes and testimonials, to enhance engagement [35].

Five studies included self-management functions [30,32,34-36]. These included features such as blood glucose tracking [30,32,36], carbohydrate counting [30,32,36], bolus calculators [30,36], and reminders or alarms [30,34].

Three studies incorporated peer support functions, such as forums and group chat features, into the mHealth and web-based interventions [30,34,35]. Additionally, 2 interventions allowed participants to message a health care professional directly [30,33,34].

Finally, 3 studies included parent-specific functions: one of these studies [33] developed a mobile-friendly web app to help parents identify and reinforce positive diabetes-related behaviors using strength-based rating. The remaining 2 studies (25%) [31,34] focused on providing parents with information on effectively supporting their teenaged children with diabetes.

**Table 3.** Physical and psychological well-being outcomes of mobile health and web-based interventions.

Study outcome measures and scales	Baseline severity	Results		Functions included in study
		Significance	*P* value	
Carreira et al (2023) [29]				Therapy
HbA_1c^a^_	64‐75 mmol/mol	NS^b^	.83	
Diabetes distress				
DDS^c^	Low diabetes distress	Significant	.02	
Depression				
BDI-FS^d^	—^e^	Significant	.002	
Fear of hypoglycemia				
FH-15^f^	—	NS	.09	
Anxiety				
STAI-S^g^	—	NS	.09	
STAI-T^h^	—	Significant	.003	
Tack et al (2018) [30]				Self-management Peer support HCP^i^-patient support
HbA_1c_	53‐64 mmol/mol	Significant	.047	
Diabetes distress				
PAID^j^	Moderate diabetes distress	NS	.11	
Fear of hypoglycemia				
HFS-WS^k^	—	NS	.89	
Singh et al (2023) [31]				Therapy Support for parents
HbA_1c_	53‐64 mmol/mol	NS	.30	
Diabetes distress				
PAID-T^l^	High diabetes distress	NS	.80	
Depression				
PHQ-9^m^	—	NS	.20	
CES-D^n^	—	NS	.80	
Drion et al (2015) [32]				Self-management
HbA_1c_	64‐75 mmol/mol	NS	—	
Diabetes distress				
PAID	Low diabetes distress	NS	—	
Hilliard et al (2020) [33]				Education Support for parents
HbA_1c_	>75 mmol/mol	NS	.57	
Diabetes distress				
PAID-T	High diabetes distress	NS	.96	
Castonsøe-Seidenfaden et al (2018) [34]				Self-management Peer support HCP-patient support Support for parents
HbA_1c_	>75 mmol/mol	NS	—	
Diabetes distress				
PAID	Low diabetes distress	NS	.13	
Xie et al (2023) [35]				Education Self-management Peer support
Fear of hypoglycemia				
HFS-II^o^	—	Significant	<.001	
Cuixart et al (2024) [36]				Self-management
HbA_1c_	53‐64 mmol/mol	NS	.20	
Diabetes distress				
DSS-S^p^	Low diabetes distress	NS	.37	

a HBA_1C_: glycated hemoglobin.

b NS: not significant.

c DDS: Diabetes Distress Scale.

d BDI-FS: Beck Depression Inventory-Fast Screen.

e Not applicable or not available.

f FH-15: Fear of Hypoglycemia 15-item scale.

g STAI-S: State-Trait Anxiety Inventory-State scale.

h STAI-T: State-Trait Anxiety Inventory-Trait scale.

i HCP: health care professional.

j PAID: Problem Areas in Diabetes scale.

k HFS-WS: Hypoglycemia Fear Survey-Worry Scale.

l PAID-T: Problem Areas in Diabetes-Teen scale.

m PHQ-9: Patient Health Questionnaire-9.

n CES-D: Center for Epidemiological Studies-Depression scale.

o HFS-II: Hypoglycemia Fear Survey II.

p DSS-S: Diabetes Distress Scale Spanish version.

### Study Outcomes

#### Psychological Well-Being

Of the 8 studies, only 2 (25%) reported significant improvements in psychological well-being [29, 35]. The first study, which used an internet-based CBT intervention targeting mild to moderate depressive symptoms, reported a reduction in diabetes distress (*P*=.02), depression (*P*=.002), and anxiety (*P*=.003), despite the intervention lasting only 9 weeks [29].

The second study, a 12-month trial of a self-guided web app designed to enhance type 1 diabetes self-management, demonstrated significant improvement in fear of hypoglycemia (*P*<.001) [35]. The functions associated with the improvements in these studies included therapy [29] and educational functions with self-management and peer-support functions [35].

#### Glycemic Control

Only 1 study [30] reported improvements in glycemic control within 6 weeks. This study involved a mobile app featuring data logging, a bolus calculator function, a peer support forum, and direct messaging with HCPs.

### Study Quality

**Figure 2.**
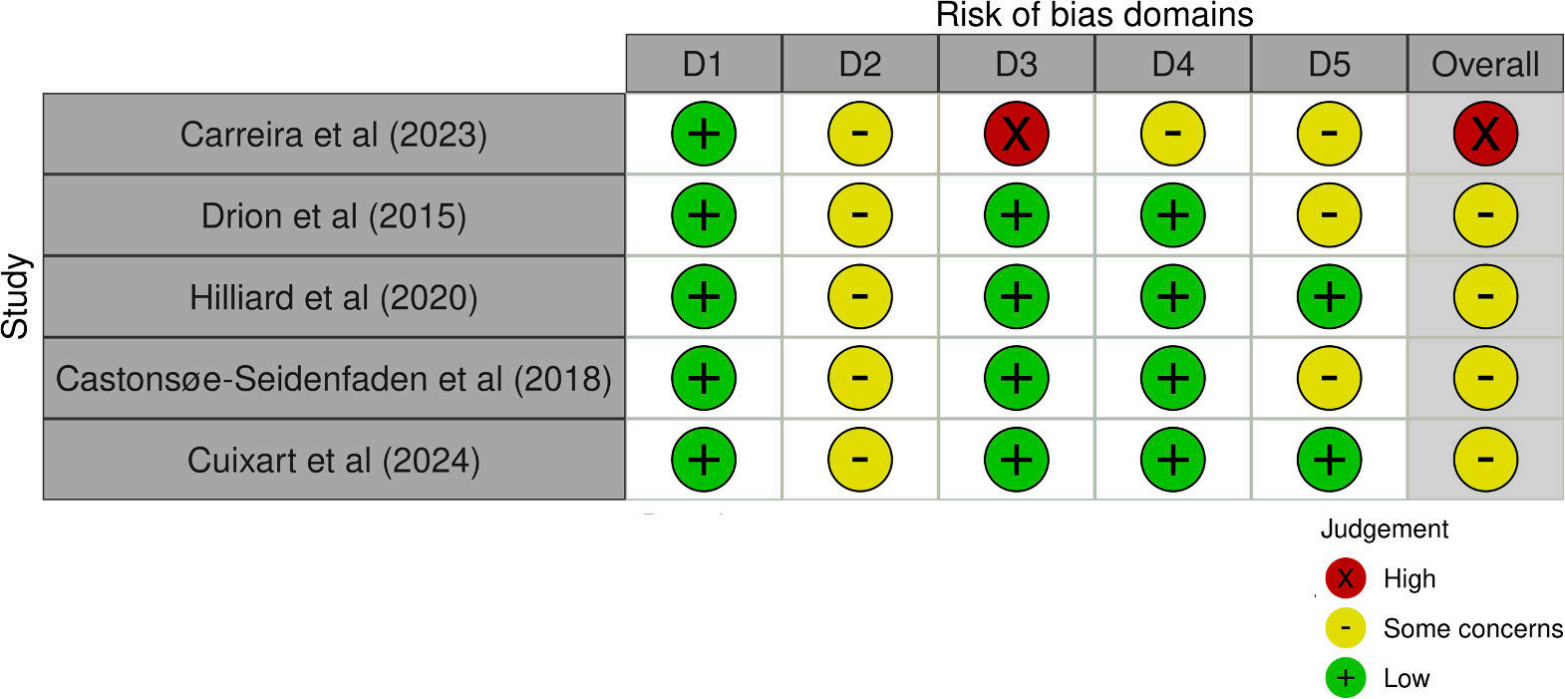
Risk of bias plot: Risk of Bias 2 tool evaluation of the 5 randomized controlled trial studies [29,32-34,36]. Domains: D1, bias arising from the randomization process; D2, bias due to deviations from intended intervention; D3, bias due to missing outcome data; D4, bias in measurement of outcomes; D5, bias in selection of the reported result.

According to the RoB 2 evaluation of the 5 RCT studies (see Figure 2), 4 studies were considered to have some concerns of bias [32-34,36]. This concern was primarily due to their inability to blind the participants to the intervention. One study was deemed to have a high risk of bias due to the high attrition rate, with 28 of the 65 participants (43%) remaining in the study [29].

**Figure 3.**
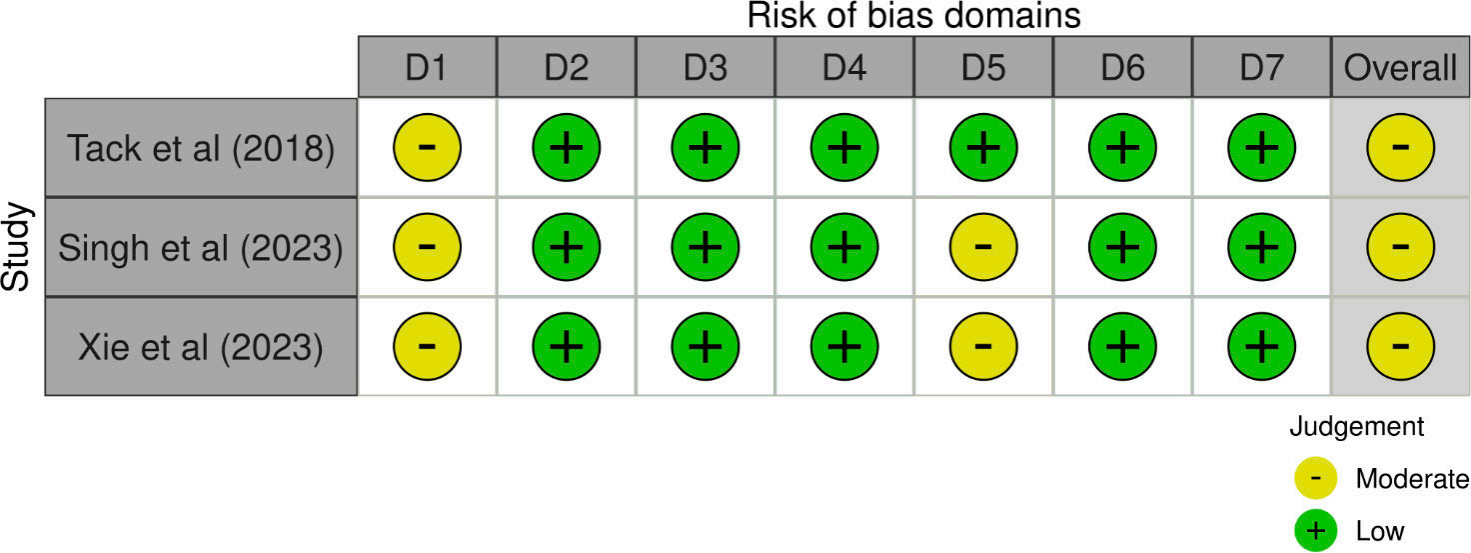
Risk of bias plot: Risk of Bias in Non-randomised Studies - of Interventions (ROBINS-I) evaluation of 3 nonrandomized intervention studies [30,31,35]. Domains: D1, bias due to confounding; bias due to selection of participants; D3, bias in classification of interventions; D4, bias due to deviations from intended interventions; D5, bias due to missing data; D6, bias in measurement of outcomes; D7, bias in selection of the reported result.

In the ROBINS-I evaluation of the nonrandomized intervention studies (see Figure 3), all 3 studies were considered to have a moderate risk of bias [30,31,35]. This was mainly due to the high attrition rates and the presence of uncontrolled confounders in each study.

## Discussion

### Principal Findings

This systematic review identified 8 studies that explored the effects of mHealth and web-based interventions on the psychological well-being of people with type 1 diabetes. Only 2 of the 8 studies (25%) reported significant positive effects on psychological well-being, indicating a low overall effectiveness rate. Nevertheless, six categories of functions used in the included interventions were identified: (1) therapy, (2) education, (3) self-management, (4) peer support, (5) HCP-patient support, and (6) parental support.

### Therapy

One of the 2 studies that demonstrated a positive impact on psychological well-being incorporated elements of therapy. This study used an internet-based CBT program that included informational content, examples, and self-assessments [29], which significantly improved the diabetes distress, depression, and anxiety levels in participants [29]. These results align with previous research highlighting the effectiveness of CBT in managing psychological well-being, including stress, anxiety, and depressive symptoms, among individuals with type 1 diabetes [37]. Incorporating theoretical frameworks, particularly CBT, into digital interventions appears to be a promising approach. The key advantages of internet-based CBT are that it is accessible and it enables users to engage with the content at their own pace. Furthermore, internet-based CBT has been associated with greater improvements in depressive symptoms [38].

In contrast, another study that combined CBT and behavioral activation components showed no significant effect on psychological well-being [31]. This may be due to the study’s inclusion of adolescents without chronic conditions, making it less relevant for addressing the daily challenges of type 1 diabetes [31]. The study participants suggested that including diabetes-related content could have improved the intervention [31]. Similarly, previous studies, including Singh et al [31]*,* have emphasized the importance of tailoring interventions to the needs of the target population and stated that the lack of such tailoring contributed to an intervention’s ineffectiveness [39].

### Education

Educational content, alongside therapy, plays an important role in type 1 diabetes interventions by enhancing knowledge about the condition and its practical management [40]. Two studies incorporated educational content delivered in distinct forms [33,35]. The first used psychoeducational videos but reported no improvement in psychological well-being [33]. This outcome may be attributed to the study design, as the study primarily targeted the parents of people with type 1 diabetes rather than individuals living with the condition. Consequently, the intervention may have failed to directly address the psychological needs and unique challenges faced by people with type 1 diabetes. Additionally, the limited benefits observed in previous studies using psychoeducation for type 1 diabetes may be attributed to several factors, including the absence of psychology specialists in intervention design, low patient engagement, and the inherent difficulty of altering established management behaviors [41,42].

In contrast, the second study incorporated diabetes self-management education (DSME) features, which helped individuals feel more empowered to manage their condition more effectively and led to reduced fear of hypoglycemia [20,21]. These improvements may be related to the participatory approach in developing the intervention, involving a broad range of type 1 diabetes stakeholders, including patients, HCPs, and family members.

### Self-Management

Self-management features, such as glucose tracking and carbohydrate counting, are the most frequently included components of mHealth apps and were the predominant feature identified in this review [43]. Most self-management features primarily support physical health and do not directly address psychological well-being, as noted by Knox et al [20]. Therefore, when psychological well-being outcomes are considered, they are often measured as secondary outcomes. This was demonstrated in 3 of the 5 studies (60%) in this review that included self-management functions, where psychological well-being was measured as a secondary outcome [32,34,36]. Hence, to fully address the needs of individuals living with type 1 diabetes, self-management features for type 1 diabetes may need to be supplemented with additional strategies that explicitly target psychological health.

### Parental Support

Three studies focused on developing interventions for children and adolescents (aged 10‐18 years) living with type 1 diabetes [31,33,34], with all 3 including parental support as a function [31,33,34]. The 3 studies highlighted the importance of maintaining a good parent-child relationship regarding the management of type 1 diabetes.

To date, parental features in mHealth and web-based interventions for type 1 diabetes remain limited and show minimal effects on psychological well-being outcomes, as support for parental features is usually secondary [31,34]. Nevertheless, one of the studies developed an intervention for parents and adolescents with type 1 diabetes [33]. While the intervention did not directly address adolescent stress factors or psychological difficulties, parent-teen communication was positively influenced.

Therefore, considering that family conflict, parenting style, support, involvement, and relationship quality are closely linked to psychological well-being among youth with type 1 diabetes [44], it is surprising that mHealth and web-based interventions rarely focus on supporting parents and their relationship with their children. This highlights the need for more targeted interventions to better support parents and their relationship with their child to help them better manage their condition [33].

### Peer and HCP-Patient Support

Finally, 3 of the 8 studies (38%) incorporated peer and HCP-patient support functions and features within mHealth and web-based apps. However, only 1 of the studies, involving a discussion forum for users to communicate with each other, found an improvement in psychological well-being. Importantly, in all 3 studies, these features were secondary to the main purpose of the app, whereas educational and self-management features were prioritized. Chat rooms, forums, and direct messages to HCPs were features that were added as support components and were not central to the intervention design. As a result, it remains difficult to determine the extent to which these support functions contributed to psychological outcomes.

Despite this, the incorporation of peer support and HCP-patient support in mHealth apps is beneficial [45]. A previous systematic review of peer support interventions showed that 5 of 8 studies using video-based telehealth improved diabetes distress or depression [46]. In addition, a recent qualitative study involving adolescents living with type 1 diabetes highlighted their interest in the potential benefits of app-based peer support for providing emotional assistance, including features such as moderated chats for safety and video calls to foster emotional connection [47].

These findings suggest that incorporating video-based features and moderated chat rooms could facilitate communication with HCPs and peers with type 1 diabetes, especially because adolescents often prefer digital interactions over in-person ones, which is also stated in one of the included studies, which found that chat rooms were the most popular feature among adolescents with type 1 diabetes [24].

### Synergy Between Functions

In total, 5 of the 8 studies (63%) included at least 2 functions within their intervention, with only 1 study reporting improvements in psychological well-being [35].

Therefore, it is difficult to attribute the effectiveness to any single function or feature within the interventions. Nevertheless, certain combinations of functions and features may have greater potential for improving psychological well-being when designed to work synergistically.

As seen in the study by Xie et al [35]*,* the inclusion of educational features may have strengthened the users’ knowledge and supported more effective use of self-management tools, such as the automated carbohydrate counter embedded within their educational content, resulting in a decrease in fear of hypoglycemia. Additionally, peer support may have offered encouragements that helped sustain positive behavioral changes over time.

While certain functions, such as peer support, may not independently improve psychological well-being, they could add value when integrated with other functions such as therapy or education by creating more engagement with the intervention and providing support.

This may be relevant to the study by Carreira et al [29], which reported improvements in psychological well-being through therapy-related functions alone. However, the study reported low adherence due to the high level of effort and motivation required [29]. Therefore, incorporating peer support functions, such as forums or chat rooms, could have helped participants remain motivated. In addition, self-management features, typically developed to improve physical outcomes such as glycemic control, may provide more holistic support when integrated with therapy functions, supporting both mental and physical health in individuals with type 1 diabetes [20]. Hence, identifying which specific combinations of features and functions are most effective in improving psychological well-being may help optimize these interventions.

### Challenges in Identifying Effective Functions

Several factors, apart from the limited number of studies, contributed to the difficulty of identifying specific functions or features within mHealth and web-based interventions. First, the intervention duration may have influenced the study outcomes. Some of the included studies were relatively short-term, with 2 studies lasting less than 3 months, limiting the participants’ ability to experience or fully sustain psychological changes. Long-term studies are more likely to capture the cumulative effects of interventions and provide insights into their lasting impact. For example, although the CBT study demonstrated significant improvements in psychological well-being over 9 weeks, it remains uncertain whether these benefits would persist without continued engagement [29].

Second, differences in the participants’ baseline characteristics, particularly the severity of diabetes distress, may have influenced the results. Most studies found no significant improvements in psychological or glycemic outcomes, regardless of the baseline diabetes distress levels. Of the 8 studies reviewed, 6 reported low to moderate diabetes distress, with only 1 reporting improvement. This suggests that individuals with lower diabetes distress may have limited potential for noticeable change, making it harder to observe significant benefits from interventions. Additionally, the variability in the diabetes distress measurement scales (PAID, PAID-Teen [PAID-T], and DDS) and follow-up periods between interventions contributed to the observed heterogeneity across studies.

Finally, as highlighted previously, 5 of the 8 studies in this review treated psychological well-being as a secondary outcome, which may have influenced the design and implementation of targeted interventions. Prioritizing psychological well-being as a primary outcome in future research could enable the development of interventions explicitly aimed at improving psychological well-being, potentially leading to more consistent and significant improvements.

Overall, identifying specific functions and features that may impact psychological well-being is challenging due to the limited number of relevant studies, variations in participant characteristics and study designs, differences in outcome measures and scales, and psychological well-being often being measured as a secondary outcome.

### Comparison to Prior Work and Future Directions

Despite the aforementioned challenges, several factors have been identified in both this review and previous work as beneficial for the development of mHealth and web-based interventions to improve psychological well-being.

Previous reviews, notably Knox et al [20] and Garner et al [21], found that digital health interventions provide some evidence for improving self-efficacy and quality of life in children, adolescents, and young adults with type 1 diabetes.

In this review, only studies that specifically measured psychological well-being were included, resulting in the inclusion of fewer studies and smaller observed improvements than those in the previous reviews [20,21]. We focused on psychological well-being, as it directly reflects the emotional burden of living with type 1 diabetes, which is important for improving self-care and related outcomes. On the other hand, many other factors may affect self-efficacy and quality of life, beyond psychological well-being. Future studies should therefore also focus on measuring psychological well-being alongside self-efficacy and quality of life to develop interventions with a direct positive impact.

In addition, the use of a participatory approach of various type 1 diabetes stakeholders in the design process is crucial. Two studies that reported improvement in psychological well-being and glycemic control, respectively, adopted a participatory approach involving patients with type 1 diabetes [30,35] along with HCPs and researchers specialized in type 1 diabetes, including a psychologist [35]. This highlights the importance of involving type 1 diabetes stakeholders in the development of an mHealth intervention, as also established by Yakubu et al [46], to ensure that the intervention is relevant, user-friendly, and aligned with patient needs.

As previously stated, future research could explore which combinations of functions and features most effectively support both psychological well-being and glycemic control, with input from type 1 diabetes stakeholders. This approach also reinforces the need to prioritize psychological well-being equally with glycemic outcomes when developing mHealth and web-based interventions to offer more holistic support. Co-designing interventions with type 1 diabetes stakeholders may also help ensure that the selected functions are both relevant and acceptable to users.

Additionally, integrating psychological and behavioral theories in intervention development appears to play an important role, as highlighted by Garner et al [21]. Future studies should ensure that mHealth and web-based interventions are developed using evidence-based psychological theories, as such interventions are reported to be more effective [46]. Given the documented efficacy of these theories in digital interventions, not using them while developing new interventions may be concerning [48]. Digital interventions incorporating theories such as social cognitive theory, the health belief model, and especially CBT have been shown to improve psychological well-being in people living with chronic conditions and mental health problems; prioritizing the use of these theories can enhance the relevance and efficacy of interventions [48,49].

Finally, tailoring the intervention to the age group of people living with type 1 diabetes is also important, as preferences for specific features may vary by age. For example, chat rooms are particularly popular among adolescents. This further highlights the need to involve relevant type 1 diabetes stakeholders in intervention design and development, to ensure that they meet user needs and preferences.

### Strengths and Limitations

A key strength of this review was the inclusion of individuals with type 1 diabetes aged 10 years and older, broadening our understanding of how mHealth and web-based interventions support psychological well-being across different age groups. This also ensures a more inclusive representation of the varied experiences and needs of the type 1 diabetes population.

The review also adhered to the PRISMA (Preferred Reporting Items for Systematic Reviews and Meta-Analyses) guidelines and followed a registered protocol, ensuring methodological transparency and adherence to reporting standards. Additionally, the clear eligibility criteria ensured the inclusion of relevant studies and the replicability of the review. Furthermore, it identified key intervention functions and features, offering practical insights to guide future app development tailored to user needs.

However, this review is not without limitations. The included studies showed significant variation in intervention type, duration, sample size, design process, and outcome measures. The heterogeneity of the studies made it challenging to identify the specific functions and features that support the psychological well-being of patients with type 1 diabetes.

Additionally, the limited number of studies included in this review, along with the differences in study duration and the use of different versions of questionnaires to measure diabetes distress, anxiety, and depression, hindered the ability to conduct a meta-analysis and introduced inconsistencies in the assessment of psychological well-being. Specifically, the use of different scales to measure diabetes distress, such as different versions of the PAID scale (PAID-20 and PAID-T), DDS, and the Spanish version of DDS (DSS-S), made meta-analysis unfeasible. Although the PAID scale and DDS are the most common diabetes distress measurement scales, they differ notably: the PAID scale addresses a broader range of emotional concerns, while the DDS reflects distress related to diabetes self-management [50]. Additionally, differences between the 2 versions of the PAID scale did not allow complex comparisons. The PAID consists of 20 items rated on a 4-point scale, whereas the PAID-T includes 26 items rated on a 6-point scale and is designed for teenagers. To improve consistency in future research, a single standardized version of the PAID instrument should be adopted, to allow comparisons and improve the validity of meta-research.

In addition, the digital interventions may benefit individuals recently diagnosed with type 1 diabetes, who have more to learn about managing the condition. However, participants in the included studies had lived with type 1 diabetes for at least 5 years, suggesting that they likely had significant experience in self-management, limiting the impact of the interventions. Lastly, excluding studies that included both type 1 diabetes and type 2 diabetes may have led to missing potentially informative data.

### Conclusion

This review highlights the limited effectiveness of mHealth and web-based interventions in improving the psychological well-being of people living with type 1 diabetes. While some interventions demonstrated promising results, the findings emphasize the need for more stakeholder involvement in intervention design and development, theory-based approaches, and potentially effective combinations of functions and features. By addressing the challenges identified in this review, future interventions can offer more comprehensive support to people living with type 1 diabetes.

## Supplementary material

10.2196/75280 Multimedia Appendix 1 Supplementary information on the search strategy, study characteristics and design, mHealth app functions and features, population characteristics, physical and psychological outcomes, and risk of bias assessment of randomized and nonrandomized studies.

10.2196/75280 Checklist 1 PRISMA Checklist.

## Abbreviations

CBT: cognitive behavioral therapy
DDS: Diabetes Distress Scale
DSME: diabetes self-management education
HCP: health care professional
mHealth: mobile health
PAID: Problem Areas in Diabetes
PAID-T: PAID-Teen
PRISMA: Preferred Reporting Items for Systematic Reviews and Meta-Analyses
RCT: randomized controlled trial
RoB 2: Risk of Bias 2 tool
ROBINS-I: Risk of Bias in Non-randomised Studies - of Interventions tool
