# Supplementary material for: Functions, Features, and Psychological Well-Being Impacts of Type 1 Diabetes Self-Management Mobile and Web Apps: Systematic Review
Source: J Med Internet Res. 2025 Nov 19;27:e75280. doi: 10.2196/75280 (PMC12658348; doi:10.2196/75280)
Supplement: Multimedia Appendix 1 [file jmir-v27-e75280-s001.docx]

**Supplementary Table S1. Search Strategy**

## Table S1a: Search strategy and results on PUBMED

| #4 | Search: #1 AND #2 AND #3 |
| --- | --- |
| #3 | Search: (("Mental Health"[Mesh]) OR "Depression"[Mesh]) OR "Anxiety"[Mesh] OR "Diabetes Distress"[tiab] OR "Depression"[tiab] OR "Fear of Hypoglycaemia"[tiab] OR "Psychosocial"[tiab] or "mental disorder*"[tiab] or "Psychological*"[tiab] |
| #2 | Search: ((("Mobile Applications"[Mesh]) OR "Telemedicine"[Mesh]) OR "Mobile App*"[tiab] OR "Mobile Health App*"[tiab] OR "mobile-based"[tiab] OR "smartphone" OR "mhealth"[tiab] |
| #1 | Search: "Diabetes Mellitus, Type 1"[Mesh] OR "Type 1 Diabete*"[tiab] |

## Table S1b: Search strategy and results on EMBASE

| 1. | Type 1 Diabetes.mp. or insulin dependent diabetes mellitus/ |
| --- | --- |
| 2. | T1DM.mp. |
| 3. | T1D.mp. |
| 4. | mobile app.mp. or mobile application/ |
| 5. | mobile phone/ or mobile health application/ |
| 6. | telemedicine/ or health app.mp. or mobile phone/ |
| 7. | smartphone/ or smartphone application.mp |
| 8. | mHealth.mp. |
| 9. | distress syndrome/ or Diabetes distress.mp. |
| 10. | depression/ |
| 11. | anxiety/ |
| 12. | psychological well-being/ |
| 13. | mental health/ |
| 14. | psychosocial.mp. |
| 15. | 1 or 2 or 3 |
| 16. | 4 or 5 or 6 or 7 or 8 |
| 17. | 9 or 10 or 11 or 12 or 13 or 14 |
| 18. | 15 and 16 and 17 |
| 19. | limit 18 to (article or article in press) |

##

## Table S1c: Search strategy and results on Web of Knowledge

| **#1** | ((((ALL=(Type 1 Diabetes)) OR ALL=(T1D)) OR ALL=(T1DM)) OR ALL=(Insulin-Dependent Diabetes Mellitus)) OR ALL=(Type 1 Diabetes Mellitus ) |
| --- | --- |
| **#2** | **(((((((ALL=(Mobile health app*)) OR ALL=(mobile app*)) OR ALL=(Smartphone)) OR ALL=(mhealth)) OR ALL=(mobile health)) OR ALL=(Telemedicine)) OR ALL=(Web-based)) OR ALL=(web)** |
| **#3** | **(((((((ALL=(Diabetes Distress)) OR ALL=(Depression)) OR ALL=(Anxiety)) OR ALL=(Fear of hypog*)) OR ALL=(Mental health)) OR ALL=(psycholog*)) OR ALL=(Wellbeing)) OR ALL=(Psychosocial)** |
| **#4** | **#3 AND #2 AND #1** |

## Table S1d: Search strategy and results on Cochrane Review

| #1 | MeSH descriptor: [Diabetes Mellitus, Type 1] this term only |
| --- | --- |
| #2 | “Type 1 Diabetes” |
| #3 | “Type 1 Diabetes Mellitus” |
| #4 | insulin dependent diabetes |
| #5 | #1 OR #2 OR #3 OR #4 |
| #6 | MeSH descriptor: [Mobile Applications] explode all trees |
| #7 | Mobile app |
| #8 | Mobile health app |
| #9 | mobile health application |
| #10 | smartphone |
| #11 | mobile-based |
| #12 | MeSH descriptor: [Telemedicine] this term only |
| #13 | mhealth |
| #14 | web-based |
| #15 | #6 OR #7 OR #8 OR #9 OR #10 OR #11 OR #12 OR #13 OR #14 OR #15 |
| #16 | MeSH descriptor: [Mental Health] explode all trees |
| #17 | mental health |
| #18 | depression |
| #19 | diabetes distress |
| #20 | anxiety |
| #21 | MeSH descriptor: [Depression] explode all trees |
| #22 | psychosocial |
| #23 | fear of hypoglycaemia |
| #24 | psychological wellbeing |
| #25 | #16 OR #17 OR #18 OR #19 OR #20 OR #21 OR #22 OR #23 OR #24 |
| #26 | #5 AND #15 AND #25 |

## Table S1e: Search strategy and results on SCOPUS

| #1 | TITLE-ABS-KEY ( "type 1 diabetes" OR "type 1 diabetes mellitus" OR t1d OR t1dm ) |
| --- | --- |
| #2 | TITLE-ABS-KEY ( "Mobile health app*" OR “mobile app*” OR "mHealth" OR "Smartphone" OR “mobile phone” OR "telemedicine" OR "mobile-based" OR "Web" OR "Web based" ) |
| #3 | TITLE-ABS-KEY ( " Diabetes Distress" OR "Depression" OR "Anxiety" OR "fear of hypog*" OR "mental health" OR "psycholog*" OR "Wellbeing" ) |
| #4 | #1 AND #2 AND #3 |

## Table S1f: SSearch strategy and results on PsychINFO

| #1 | (T1D* or Type 1 Diabetes or Type 1 Diabetes Mellitus).af. |
| --- | --- |
| #2 | (smartphone or Web based or Web or Mhealth or telemedicine* or mobile app* or mobile health app*).af. |
| #3 | (Diabetes Distress or Depression or Anxiety or Fear of Hypo* or psycholog* or wellbeing or mental health).af. |
| #4 | #1 AND #2 AND #3 |

# Supplementary Table S2a. Data Extraction Table – Study Information:

| **Study Information** | | | | |
| --- | --- | --- | --- | --- |
|  | **Study Title** | **Authors** | **Year of Publication** | **Country** |
| 1. **Carreira et al. (2023) [29]** | Internet-based cognitive-behavioural therapy is effective in reducing depressive symptomatology in type 1 diabetes: results of a randomized controlled trial | Mónica Carreira , Ma Soledad Ruiz de Adana , José Luis Pinzón , María Teresa Anarte-Ortiz , | 2023 | Spain |
| 1. **Tack et al. (2018) [30]** | Glucose Control, Disease Burden, and Educational Gaps in People with Type 1 Diabetes: Exploratory Study of an Integrated Mobile Diabetes App | Cornelis J Tack, Gerardus J Lancee, Barend Heeren, Lucien JLPG Engelen, Sandra Hendriks, Lisa Zimmerman, Daniele De Massari, Marleen MHJ van Gelder, Tom H van de Belt. | 2018 | Netherlands |
| 1. **Singh et al. (2023) [31]** | Internet-based cognitive behavioural therapy intervention for youth with type 1 diabetes and depressive symptoms: a pilot and feasibility study | Singh, P., Otero, J. M., Howe, C., Feinstein, R. T., Gupta, K., Gladstone, T. R. G., Van Voorhees, B. W., Gupta, O. T. | 2024 | USA |
| 1. **Drion et al. (2015) [32]** | The effects of a mobile phone application on quality of life in patients with type 1 diabetes mellitus: A randomized controlled trial | Drion, I.; Pameijer, L. R.; Van Dijk, P. R.; Groenier, K. H.; Kleefstra, N.; Bilo, H. J. G. | 2015 | Netherlands |
| 1. **Hilliard et al. (2020) [33]** | Type 1 Doing Well: Pilot Feasibility and Acceptability Study of a Strengths-Based mHealth App for Parents of Adolescents with Type 1 Diabetes | Marisa E. Hilliard, PhD, Viena T. Cao, BS, Sahar S. Eshtehardi, MS,  Charles G. Minard, PhD, Rana Saber, MS, MSL, Debbe Thompson, PhD,  Lefkothea P. Karaviti, MD, PhD, and Barbara J. Anderson, PhD | 2020 | USA |
| 1. **Castonsøe-Seidenfaden et al. (2018) [34]** | Testing a Smartphone App (Young with Diabetes) to Improve Self-Management of Diabetes Over 12 Months: Randomized Controlled Trial | Pernille Castensøe-Seidenfaden, MD, PhD,corresponding author Gitte Reventlov Husted, RN, MScN, PhD Andreas Kryger Jensen, PhD Eva Hommel, MD, DMSc, Birthe Olsen, MD, Ulrik Pedersen-Bjergaard, MD, DMSc, Professor, Finn Kensing, DSc, Professor, and Grete Teilmann, MD, PhD | 2018 | Denmark |
| 1. **Xie et al. (2023) [35]** | Evaluation of Support, a self-guided online type 1 diabetes self-management education and support web application—a mixed methods study | Li Feng Xie, Asmaa Housni, Amélie Roy-Fleming , Aude Bandini, Treena Delormier, Deborah Da Costa, Anne-Sophie Brazeau | 2023 | Canada |
| 1. **Cuixart et al. (2024) [36]** | Can a mobile application improve glucose-related and patient-reported outcome measures (PROMs) in people with type 1 diabetes mellitus? A randomized controlled trial using the mySugr app | Gemma Cuixart, Rosa Corcoy,Cintia González | 2024 | Spain |

#

# Supplementary Table S2b. Data Extraction Table – Study Design

| **Study Design** | | | | |
| --- | --- | --- | --- | --- |
|  | **Study Type** | **Inclusion** | **Exclusion** | **Psychological Outcome Classification** |
| 1. **Carreira et al. (2023) [29]** | RCT | - Medical diagnosis of type 1 Diabetes for ≥1 year - Over 18 years old - Mild to moderate depressive symptoms - No pharmacological treatment affecting blood glucose or depressive symptoms - No current psychological treatment - Absence of: Chronic renal failure, Impaired liver function, active thyroid disease (except correctly managed hypothyroidism), current pregnancy, acute ketosis decompensation at study starts - Internet access | - Type 2 diabetes - Women who are pregnant or planning pregnancy - Severe macrovascular or microvascular complications - No depressive symptoms or severe depressive symptoms - Diagnosis of major depressive disorder with suicide risk - Non-collaboration (no signed informed consent) - Disabling psychiatric disorder - Psychosis - No internet access | Primary |
| 1. **Tack et al. (2018) [30]** | Pre-Post study | - Adults with type 1 diabetes - 18-65 years old - Diabetes duration of 2 years - Stable HbA1c (between 7-10%) - Able to count carbohydrates and bolus insulin - Having a body mass index between 18 and 35 kg/m² - Using a suitable mobile phone or tablet - Speak and read in Dutch | - - People with serious type 1 diabetes complications   - Severe retinopathy with poor vision (visual acuity <0.5)   - Renal failure (glomerular filtration rate <30 mL/min/1.73 m²)   - Foot amputation   - Recent (<6 months) myocardial infarction or stroke   - Any serious comorbidity deemed to significantly affect participation   - A history of severe hypoglycaemia (requiring third-party assistance) over the past 3 years   - Pregnancy or aiming for pregnancy   - Total insulin needs greater than 1 U/kg/day | Primary |
| 1. **Singh et al. (2023) [31]** | Pre-Post study | Adolescents 13 to 17 years old with type 1 diabetes and with mild (score 5–9) or moderate (score 10–14) depressive symptoms on a Patient Health Questionnaire-Adolescent (PHQ-A) | - Patients who were medically unstable (i.e., current episode of DKA, symptomatic hypoglycaemia), - PHQ-A ≥20 - Diagnosis of schizophrenia or bipolar disorder, prior psychiatric hospitalization - Prior self-harm attempt - Participating in ongoing counselling or therapy services in the last year by a licensed professional (counsellor, psychologist, or psychiatrist) - Currently taking psychotropic medications. | Primary |
| 1. **Drion et al. (2015) [32]** | RCT | - 18 years old with type 1 diabetes - Treated with multiple daily injections (MDI), continuous subcutaneous insulin infusion (CSII), or continuous intraperitoneal insulin infusion (CIPII). | - Used a diabetes application in the 3 months prior to their visit - Did not have internet or email access - Were unable to read Dutch. | Primary |
| 1. **Hilliard et al. (2020) [33]** | RCT | - Adolescent age 12–17 years at enrolment - Type 1 diabetes duration of at least 6 months - Receiving care at Texas Children’s Hospital - Fluency in English | - Parent or adolescent with a serious medical, cognitive, or mental health comorbidity that would preclude their ability to provide informed consent or participate in data collection or intervention - Families were not eligible to participate in the trial if the parent did not have a smartphone with Internet access/data plan - Three families were ineligible based on this criterion | Secondary |
| 1. **Castonsøe-Seidenfaden et al. (2018) [34]** | RCT | - Type 1 diabetes for more than one year - Received diabetes care - Age between 4-22 - HbA1c ≥64 mmol/mol (8%) at their last visit and an average HbA1c >58 mmol/mol (7.5%) at the last three visits prior to invitation - Did not attend appointments with a psychiatrist or psychologist - Spoke and understood Danish - Did not participate in other diabetes intervention studies | N/A | Secondary |
| 1. **Xie et al. (2023) [35]** | Mixed-Methods study | - Adults (≥ 18 years old) - A self-reported diagnosis of type 1 diabetes ≥ 1 year, and daily use of ≥ 4 insulin injections or an insulin pump - Access to the Internet, an active e-mail address Understanding English or French. | - People with ongoing pregnancy or illnesses limiting diabetes care or limiting access to educational tools (e.g., dementia and blindness) | Primary |
| 1. **Cuixart et al. (2024) [36]** | RCT | - Adults (≥ 18 years) - Type 1 diabetes with duration > 1 year - Basal-bolus insulin regimen with multiple daily injections - Last (< 3 months) HbA1c ≥ 53 mmol/mol (7%) and < 75 mmol/mol (9%) - Knowledgeable about carbohydrate counting - Functional insulin treatment, - Regular use of smartphone or tablet (android/iOS). | - Use of an application for diabetes management at study entry, utilisation of real-time or intermittent continuous glucose monitoring - Being pregnant or planning pregnancy and having any disease or clinical condition that might interfere with the study protocol (e.g., active cancer, severe mental disorder, or planning for surgery). | Secondary |

# Supplementary Table S2c. Data Extraction Table – mHealth applications: Functions and Features

| **mHealth applications: Functions and Features** | | | | | | | | |
| --- | --- | --- | --- | --- | --- | --- | --- | --- |
|  | **Intervention** | | **Functions and Features** | | | | | |
|  | *Name* | *Aim* | *Therapy* | *Education* | *Self-Management* | *Peer-Support* | *HCP-Patient Support* | *Support for Parents* |
| 1. **Carreira et al. (2023) [29]** | WEB_TDDI1 | To administer an Internet-based CBT program designed and developed by our research group for the treatment of mild-moderate depressive symptomatology in individuals with type 1 diabetes and to evaluate the efficacy of this program. | The web program includes nine weekly sessions based on CBT. The topics were: Depression. Diabetes and depression, Stress and diabetes, Coping in diabetes, Resolution of problems, Pleasurable activities, Cognitive restructuring, social skills, Importance of support, relapse prevention |  |  |  |  |  |
| 1. **Tack et al. (2018) [30]** | N/A | To evaluate a prototype integrated mobile phone diabetes app in people with type 1 diabetes designed to support self-management, decrease disease burden, and benefit diabetes control. |  |  | • **Logbook to capture key measurements:** Users can manually enter blood glucose levels, as well as hypoglycaemic events, carbohydrate intake, injected insulin dose, expected physical activity, stress, and mood.  • **Carbohydrate intake data entry support**: the “meal picker” provides a means to define personal standard meals and to look up carbohydrate contents of frequently used ingredients.  • **Custom settings:** Users (or their health care providers) can set a target blood glucose level, alarms as reminders, and settings for the bolus calculator (e.g., ratios), as well as an on-off switch for a warning if blood glucose value entries exceed individual limits.  • **Insulin bolus advice:** based on data entered and personal settings, such as the personal carbohydrate to insulin ratio. |  | • **Secure communication:** people with diabetes can communicate with health care providers through a secure connection.  • **Online community:** people with diabetes can connect with their peers. |  |
| 1. **Singh et al. (2023) [31]** | CATCH-IT | To assess the efficacy of Competent Adulthood Transition with Cognitive Humanistic and Interpersonal Teaching (CATCH-IT), an internet-based cognitive behavioural therapy intervention, in adolescents with type 1 diabetes and depressive symptoms. | It contains 14 online self-directed modules (five printed modules that described the CATCH-IT modules). |  |  |  |  | • Contains information for parents to support the adolescent |
| 1. **Drion et al. (2015) [32]** | DBEES | To investigate whether the use of the Diabetes Under Control (DBEES) mobile phone application, a digital diabetes diary, results in a change in quality of life for patients with type 1 diabetes mellitus (T1DM) compared with the standard paper diary. |  |  | Patients could enter diabetes-related self-care data: blood glucose values, carbohydrate intake, medication, physical exercise, and notes. Patients in the control group kept their paper diary. |  |  |  |
| 1. **Hilliard et al. (2020) [33]** | Type 1 Doing Well | To evaluate the feasibility and acceptability of Type 1 Doing Well, a new, strengths-based mHealth app for parents of adolescents with T1D. |  | Psychoeducational videos: 3 brief videos featuring psychologist for parents: Why Praise Matters; What to Look For; How To Praise A Teen. |  |  | **Text your teen:** List of messages for parents to copy and paste into a text message and personalize to praise their teens. | **Rate my Teen:** List of 16 strengths behaviours for parents to mark if their teen engaged that day, plus a free text option.  **Diabetes Strengths: Text** Description of Diabetes strengths  **Teen’s top strengths:** List of teen’s most frequent 3 strengths over the previous week, as calculated from parents' ratings |
| 1. **Castonsøe-Seidenfaden et al. (2018) [34]** | Young with Diabetes (YWD) | To Assess whether the YWD app improved young people’s self-management measured by HbA1c and three self-reported psychometric scales. |  |  | *My Department* provides information about the diabetes department  • ‘Carbohydrate counting’ provides information on how to count carbohydrates  • ‘Information about…’ provides information about multiple T1DM-topics, such as obtaining a drivers’ license  • Tips-Packages enables users to receive daily T1DM tips. (e.g. alcohol, Ramadan, hypoglycaemia)  • Reminder Function allows users to set reminders for self-management tasks." |  | • *My Page* enables users to contact their health care provider and write notes •*Chat Room* is an opportunity to chat with peers | •*To Parents* provides parents with information about how to support their teen |
| 1. **Xie et al. (2023) [35]** | SUPPORT | Evaluate users’ satisfaction with Support and investigate changes in self-reported frequency of-, fear of- hypoglycaemia, and diabetes-related self-efficacy. |  | Six categories of learning modules with several courses in each.  Contains videos, Quizzes testimonials and PDFs | Automated Calculator | Discussion Forum |  |  |
| 1. **Cuixart et al. (2024) [36]** | MySugr | To examine whether mySugr, an app for diabetes management, together with teleconsultations, can have a positive impact on these factors and, thereby, replace current clinical care. |  |  | Diabetes diary with automatic collection and analysis of data on glycemia (including estimation of HbA1c) and the possibility to manually enter information on food intake, physical activity, and insulin dose • Bolus calculator • Reminders  • Save pictures of food consumed |  |  |  |

# Supplementary Table S2d. Data Extraction Table – Population Characteristics

| **Population Characteristics** | | | | | | | |
| --- | --- | --- | --- | --- | --- | --- | --- |
|  | **Sample size** | **Study Duration** | **Control Group** | **Participants age** | **Gender Distribution (%)** | **Ethnicity Breakdown** | **Diabetes Duration (years)** |
| 1. **Carreira et al. (2023) [29]** | n = 65 | 9 weeks | Usual Patient Care | Treatment group: 37.5(11.0)  Control group: 35.0 (12.9) | 38(58) | N/A | 18.71(12.72) |
| 1. **Tack et al. (2018) [30]** | n=19 | 6 weeks | Usual Patient Care | 43.8(14.1) | 12(63) | N/A | 22.8(14) |
| 1. **Singh et al. (2023) [31]** | n=7 | 3 months | Usual Patient Care | 15.1(1.2) | (6)86 | Caucasian (71%), African American(29%) | 6.0(3.4) |
| 1. **Drion et al. (2015) [32]** | n = 63 | 3 months | Usual patient care | 33 [21] | 23(37) | N/A | 17 [16] |
| 1. **Hilliard et al. (2020) [33]** | n=80 | 16 weeks | Usual patient care | 15.3 (1.5) | 47(59) | Non-Hispanic white (61%)  Non-Hispanic black (13%)  Hispanic (19%)  Other or more than one (6%) | 5.7(3.4) |
| 1. **Castonsøe-Seidenfaden et al. (2018) [34]** | n=151 | 12 months | Usual patient care | 17.6(2.6) | 81(56) | N/A | 8.0(4.5) |
| 1. **Xie et al. (2023) [35]** | n=207 | 12 months | Usual patient care | 49.3(13.8) | 135(65) | White(96%). Other(4%) | 25.2(14.7) |
| 1. **Cuixart et al. (2024) [36]** | n = 25 | 12 months | Usual patient care | 44.5(14.8) | 12(48) | N/A | 21.3(14.1) |

# Supplementary Table S2e. Data Extraction Table – HbA1c

| **HbA1c** | | | | | | |
| --- | --- | --- | --- | --- | --- | --- |
|  | **Measurement Timepoints** | **Baseline HbA1c** | | **Follow-up HbA1c** | | **P-value** |
|  |  | **Control** | **Intervention** | **Control** | **Intervention** |  |
| 1. **Carreira et al. (2023) [29]** | 9 weeks | 8.31(1.48) | 8.22 (1.51) | 8.46 (1.60) | 7.90 (1.40) | 0.832 |
| 1. **Tack et al. (2018) [30]** | 6 weeks |  | 62.3(7.8) |  | 59.8(7) | 0.047 |
| 1. **Singh et al. (2023) [31]** | 3 months |  | 8.2(1.8) |  | 7.8(1.6) | 0.3 |
| 1. **Drion et al. (2015) [32]** | 3 months | 62 | 61 | 63 | 63 | N/A |
| 1. **Hilliard et al. (2020) [33]** | 16 weeks | 8.7(2.1) | 9.1(2.1) | 8.4(1.4) | 8.7(1.7) | 0.57 |
| 1. **Castonsøe-Seidenfaden et al. (2018) [34]** | 2, 7 and 12 months | 76.2 (14.9) | 81.1 (18.0) |  | Difference between groups:  • 12 months = 74.6 | N/A |
| 1. **Xie et al. (2023) [35]** | 12 months | • 7% or less = 41% • 7.1 - 8% = 42% • 8.1 or more = 17% |  | N/A | N/A | N/A |
| 1. **Cuixart et al. (2024) [36]** | 12 months | 58(2) | 58(2.2) | 57.45 | 55.81 | 0.202 |

# Supplementary Table S2f. Data Extraction Table – Psychological Outcomes

| **Psychological Outcomes** | | | | | | | | | |
| --- | --- | --- | --- | --- | --- | --- | --- | --- | --- |
|  | **Psychological Outcome** | **Validated Tool Measurement** | **Measurement Timepoints** | **Baseline Psych. Outcomes** | | **Baseline Severity Psych. Outcome** | **Follow-up Psych. Outcomes** | | **P-value** |
|  |  |  |  | ***Control*** | ***Intervention*** |  | ***Control*** | ***Intervention*** |  |
| 1. **Carreira et al. (2023) [29]** | Diabetes Distress  Depression  Anxiety | DDS  BDI-FS  FH-15  STAIT-T  STAIT-S | 9 weeks | 2.44 (0.95)  6.96 (1.92)  39.30(13.10)  77.76 (20.10)  54.46 (26.62) | 2.61(0.94)  6.54 (2.04)  35.77 (11.15)  74.23 (25.07)  62.94 (27.82) | above 3 = high DD. Baseline is below. mild-moderate depressive symptoms | 2.36 (1.16)  4.60 (3.06)  37.10 (12.98)  66.50 (27.07)  48.95 (30.09) | 1.48 (0.40)  0.62 (1.41)  29.87 (9.23)  77.76 (20.10)  23.75 (30.36) | 0.022  0.002  0.093  0.003  0.093 |
| 1. **Tack et al. (2018) [30]** | Diabetes Distress  Anxiety | PAID  HFS-Worry Scale | 6 weeks |  | 20.0(14.9)  25.4(6.4) | Below 40 (PAID) |  | 17.2(14.8)  25.3(7.0) | 0.11  0.89 |
| 1. **Singh et al. (2023) [31]** | Diabetes Distress  Depression  Depression | PAID-T  PHQ-9  CES-D | 3 months |  | 89.9(28.7)  8.3(2.7)  27.6(9.4) | High DD. mild (score 5–9) or moderate (score 10–14) depressive symptoms on a PHQ-A. For PHQ-9 anything above 10 is considered indicator of depression. Baseline was below |  | 88.1(35.4)  7.6(3.6)  26.3(11.8) | 0.8  0.2  0.8 |
| 1. **Drion et al. (2015) [32]** | Diabetes Distress | PAID | 3 months | 14 | 14 | below 40 | 15 | 14 | N.S |
| 1. **Hilliard et al. (2020) [33]** | Diabetes Distress | PAID-T | 16 Weeks | 63.5(29.8) | 63.5(25.8) | high | 65.2(25.7) | 65.0(26.1) | 0.96 |
| 1. **Castonsøe-Seidenfaden et al. (2018) [34]** | Diabetes Distress | PAID | 2,7 and 12 months | 24.0 (16.1) | 26.7 (19.3) | Below 40 (PAID) |  | 21.04, 22.72, 20.54  (Control versus YWDa, mean difference)_0 | 0.13 |
| 1. **Xie et al. (2023) [35]** | Fear of Hypoglycaemia | HFS-II | 6 months and 12 months | 33.8(16.5) |  |  |  | • 6 months = 31.8(15.5) •12 months = 23.6 (11.3) | • 6 months = 0.030 •12 months = < 0.001 |
| 1. **Cuixart et al. (2024) [36]** | Diabetes Distress | DSS-S | 12 Months | 2.08 (1.02) | 1.91(0.55) |  | Change after 12 months: -3.00 (-9.75, 3.25) | Change after 12 months: -2.00 (-6.00, 7.00) | 0.373 |
